# Supplementary material for: Public Health Awareness on Bat Rabies among Bat Handlers and Persons Residing near Bat Roosts in Makurdi, Nigeria
Source: Pathogens. 2022 Aug 26;11(9):975. doi: 10.3390/pathogens11090975 (PMC9505307; doi:10.3390/pathogens11090975)
Supplement: Supplementary file 1 [file pathogens-11-00975-s001.zip › pathogens-1848220-supplementary.pdf]

Supplementary material

QUESTIONNAIRE TO ASSESS THE LEVEL OF KNOWLEDGE ON BATS AND THEIR DISEASE CARRYING POTENTIAL AMONG BAT HUNTERS, BAT MEAT CONSUMERS AND PERSONS RESIDING NEAR BAT ROOSTS IN MAKURDI BENUE STATE NIGERIA.

INSTRUCTION: Please tick the options as appropriate.

Questionnaire:

The information obtained from this study will be used strictly for academic purposes only and absolute confidentiality will be ensured.

DEMOGRAPHIC INFORMATION OF RESPONDENT

1. Location(town) ______________________
2. Age (year) <19[ ] 20-30[ ] 31-40[ ] >40 [ ]
3. Marital status: Single [ ] Married [ ]
4. Gender: Male [ ] Female[ ]
5. Occupation: Unemployed [ ] Civil servant [ ] Businessman/woman[ ] Farmer [ ] Hunter [ ] Others ____________
6. Tribe: ___________
7. Level of Education: [ ] Primary [ ] Secondary [ ] Tertiary [ ] Informal
8. Religion: Christian [ ] Islam [ ] Others________________

ASSOCIATION WITH BATS

9. Have you had any contact with bats? Yes[ ] No[ ]
10. For how long have you been handling bats?
 1-5years [ ] 6-10years [ ] 11-15years [ ] greater than 15years [ ]
11. What do you wear when handling bats?
 Use bare hands [ ] Overall/lab. Coat [ ] Boots [ ] Hand gloves [ ]
 Face/nose mask [ ] None of the above [ ]
12. What is the nature of your association with bats?
 Bat Hunter [ ] Bat meat consumer [ ] Reside near bat roost [ ] Researcher [ ] Other ____________________
13. On average how many bats do you come in contact with in a day? Less than 5 [ ] 5-10 [ ] 10-15 [ ] 15-20 [ ] Greater than 20[ ] Others __________________
14. Have you ever been bitten by a bat? Yes[ ] No[ ] if no, proceed to question 16.
15. What did you do after the bite?
 Got first aid treatment [ ] Sought medical care [ ] Washed the bite with plenty of water and soap for at least 5minutes [ ] Got anti tetanus shot [ ] Treated the bite with traditional medicine [ ] Did nothing [ ]
 Others____

KNOWLEDGE

16. Have you heard of Rabies? Yes[ ] No[ ] I don't know [ ]
17. Rabies affects only animals. Yes[ ] No[ ] I don't know [ ]

18. How is rabies transmitted__________________________
19. Do bats carry disease causing pathogens? Yes[ ] No[ ] I don't know [ ]
20. What pathogens do bats carry?
    Viruses [ ]
    Bacteria [ ]
    Protozoans [ ]
    Parasites [ ]
    All of the above [ ]
    None of the above [ ]
    Other _________________________
21. Can bats transmit disease pathogens (in 20 above) to other animals? Yes[ ] No[ ]
    I don't know [ ]
22. Can bats transmit disease pathogens (in 20 above) to humans? Yes[ ] No[ ] I don't know [ ]
23. Can bats transmit rabies to humans and other animals? Yes[ ] No[ ] I don't know [ ]
24. How do bats transmit diseases to Humans? _________________________
